# Optimizing patient positioning for exoscopic cranial surgery

**DOI:** 10.3171/2025.3.FOCVID2524

**Published:** 2025-10-01

**Authors:** Paul M. Harary, David J. Park, Steven D. Chang, Michael Schulder

**Affiliations:** Stanford University School of Medicine, Stanford, CA; North Shore University Hospital, Donald and Barbara Zucker School of Medicine at Hofstra/Northwell, Manhasset, NY

TO THE EDITOR: We viewed with great interest the operative video by Sáez-Alegre et al.^1^ demonstrating how the exoscope may be adapted for keyhole approaches (Sáez-Alegre M, Ríos-Vicil C, Piper K, et al. Feasibility of exoscopic keyhole surgery: case series. *Neurosurg Focus Video*. 2024;10[1]:V3). We commend the authors for their presentation of a range of posterior fossa surgeries performed using the exoscope, which we agree offers several advantages in comparison with the operating microscope and endoscope in this setting. As noted by the authors, however, there remain challenges in the use of an exoscope, namely its positioning and manipulation, that may hinder its broader adoption. We propose that exoscopic cranial surgeries may be further improved by reconsidering traditional patient positioning to address these issues.

Sáez-Alegre et al. described the learning curve associated with exoscope use as a disadvantage relative to the conventional operative microscope. This discussion remains highly relevant since the publication of their article, given the continued evolution of exoscopic neurosurgery. Importantly, the authors report that this learning curve was the most commonly cited reason for intraoperative conversion from exoscope to microscope among surgeons at their institution. This is consistent with the literature for cranial exoscopic surgery, with several studies documenting a > 30% exoscope-to-microscope conversion rate.^2–4^ Neurosurgeons are accustomed to using the operating microscope, often with the craniotomy site tilted between 30° and 45° from the floor for ergonomic access (Fig. 1A). However, in exoscopic surgery, this positioning is often suboptimal. The exoscope is positioned immediately adjacent to, or even behind, the head of the surgeon (Fig. 1B and D). This may not only increase the risk of contamination but also contribute to difficulty maneuvering.

**FIG. 1.**
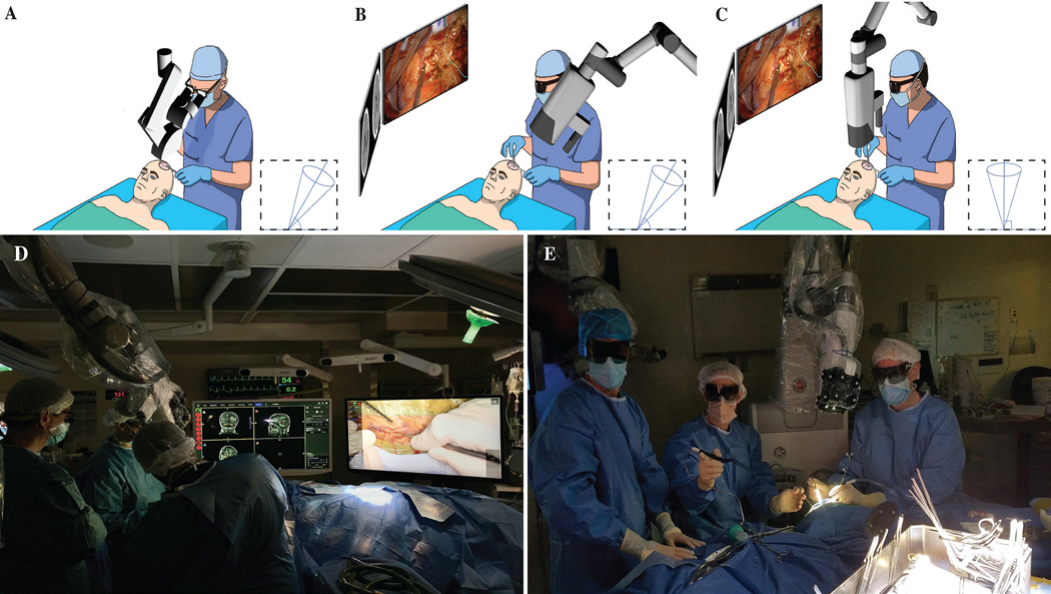
Traditional operating microscope placement for cranial surgery tilts the craniotomy site 30**°**–45° from the floor. **B and D:** In current practice, the exoscope is commonly positioned immediately adjacent to, or even behind, the head of the surgeon. **C and E:** Positioning the craniotomy as close to parallel to the floor as possible may allow the exoscope to be placed directly in front of the surgeon.

We propose implementing lessons from spine surgery, in which the patient is in a prone position and the exoscope is situated centrally between the primary and assistant surgeons. This maintains the exoscope visible to both surgeons, prevents unintended contact that could lead to contamination, and maintains optimal visualization.^5,6^ We suggest reconsidering the traditional patient positioning for exoscopic cranial surgery. Instead of tilting the craniotomy site 30°–45° from the floor, positioning it as close to parallel as possible would allow the exoscope to be placed directly in front of the surgeon, much like in spine cases (Fig. 1C and E). This adjustment has the potential to enhance visualization and ergonomics, allowing the surgeon to work with greater freedom. Furthermore, such positioning may reduce CSF drainage and brain shift, thereby improving surgical outcomes.^7^ Additionally, this may minimize risk of exoscope contamination from unintended contact. More broadly, this setup may encourage a more adaptable approach to positioning, rather than strict adherence to microsurgical conventions.

The exoscope is likely to become an increasingly common tool in cranial surgeries, particularly given recent evidence suggesting similarities in complication rates and procedural quality to the operating microscope.^8,9^ In addition, continued improvements in illumination, such as those presented by Sáez-Alegre et al., represent key progress toward addressing an early drawback of the exoscope platform.^5^ Therefore, we believe it is valuable to foster dialogue on optimal patient positioning to maximize advantages in surgeon ergonomics, visualization, and communication that the exoscope may offer.

## Disclosures

Dr. Schulder reported personal fees from Elekta and Varian outside the submitted work.
