# Supplementary material for: Optimizing patient positioning for exoscopic cranial surgery
Source: Neurosurg Focus Video. 2025 Oct 1;13(2):V20. doi: 10.3171/2025.3.FOCVID2524 (PMC12533504; doi:10.3171/2025.3.FOCVID2524)
Supplement: Related texts

# Reply: Response

**DOI:** 10.3171/2025.6.FOCVID2530

**Published:** 2025-10-01

**Authors:** Miguel Sáez-Alegre, Christian Ríos-Vicil, Keaton Piper, Edinson Najera, Walter C. Jean

**Affiliations:** Mayo Clinic, Rochester, MN; General Hospital of Castellón, Castellón de la Plana, Spain; Faculty of Biomedical and Health Sciences, Universidad Europea de Madrid, Spain; University of Puerto Rico, San Juan, Puerto Rico; Lehigh Valley Fleming Neuroscience Institute, Allentown, PA; Morsani College of Medicine, University of South Florida, Tampa, FL

## Response

We sincerely thank Harary et al. for their thoughtful comments regarding our operative video illustrating how the exoscope may be adapted for keyhole approaches. Our aim was to provide practical insights into the adaptation of the exoscope by illustrating its application in keyhole approaches to the posterior fossa. We are pleased to see the discussion it has generated.

One of the main advantages of exoscopic surgery is improved surgeon ergonomics.^1^ We fully agree with the authors’ proposal that exoscopic cranial procedures may be further enhanced by rethinking traditional patient and exoscope positioning. As shown in our video, the setup used for pineal region surgery represents a significant shift in exoscope positioning compared with the microscope, resulting in reduced fatigue and greater comfort for the surgeon. This can ultimately translate into higher surgeon satisfaction and improved surgical outcomes.^2^

As Harary et al. highlighted, the learning curve remains one of the main limitations to the widespread implementation of the exoscope in cranial surgery.^3^ A significant part of this learning curve involves identifying the optimal placement of the exoscope. When positioned too close to the surgical field, the exoscope can obstruct instrument maneuverability or provide insufficient illumination—both of which are common reasons for intraoperative conversion to the microscope.^4^ Conversely, placing the exoscope too close to the surgeon’s head may inadvertently increase the risk of contamination, potentially raising the likelihood of surgical site infections. The modifications proposed by Harary et al. directly address these challenges and additionally offer a practical solution to another technical limitation: some exoscope models allow the assistant monitor to rotate only 180°. By placing the exoscope in a face-to-face arrangement—similar to the setup commonly used in spine surgery—the assistant surgeon can maintain full visual access and actively participate in the procedure.

However, we believe that tilting the patient’s head 30°–45° can have a beneficial effect on brain relaxation. A clear example of this is during a subfrontal approach via a supraorbital craniotomy, where gravitational retraction facilitates access without the need for fixed retractors.^5^ Additionally, elevating the head of the bed by 30°–45° improves venous drainage,^6^ which contributes to a more relaxed brain. While this may indeed result in brain shift, we do not consider this to be a drawback but rather a useful physiological effect in many cases. For instance, brain shift can be disadvantageous when aiming for precision in tumor resection guided by preoperative imaging. However, in skull base procedures, brain shift is often desirable as it facilitates access to deep-seated structures while minimizing manipulation. Therefore, patient positioning should be tailored not only to the anatomical region but also to the surgical objective and strategy.

We appreciate the opportunity to engage in this dialogue and believe that continued exchange of experiences will refine best practices in exoscopic neurosurgery. We look forward to further contributions in this evolving field.

## Acknowledgments

We used ChatGPT to review the English grammar.

## References

[1] HadjipanayisCG InfingerLK LeheckaM PradillaG ZadaG. Introduction. Use of the exoscope in neurosurgery Neurosurg Focus Video 2024 10 1 V1

[2] SchupperAJ HrabarchukEI McCarthyL HadjipanayisCG Improving surgeon well-being: ergonomics in neurosurgery World Neurosurg 2023 175 e1220 e1225 37427701 10.1016/j.wneu.2023.04.102

[3] MuscasG BattistaF BoschiA MoroneF Della PuppaA A single-center experience with the Olympus ORBEYE 4K-3D exoscope for microsurgery of complex cranial cases: technical nuances and learning curve J Neurol Surg A Cent Eur Neurosurg 2021 82 5 484 489 33690881 10.1055/s-0040-1719106

[4] MontemurroN ScerratiA RicciardiL TrevisiG The exoscope in neurosurgery: an overview of the current literature of intraoperative use in brain and spine surgery J Clin Med 2021 11 1 223 35011964 10.3390/jcm11010223PMC8745525

[5] LiJ GelbAW FlexmanAM JiF MengL Definition, evaluation, and management of brain relaxation during craniotomy Br J Anaesth 2016 116 6 759 769 27121854 10.1093/bja/aew096

[6] RozetI VavilalaMS Risks and benefits of patient positioning during neurosurgical care Anesthesiol Clin 2007 25 3 631 653 x 17884711 10.1016/j.anclin.2007.05.009PMC2265668
